# Supplementary material for: One-Step, Low-Cost, Operator-Friendly, and Scalable Procedure to Synthetize Highly Pure N-(4-ethoxyphenyl)-retinamide in Quantitative Yield without Purification Work-Up
Source: Molecules. 2022 Jun 6;27(11):3632. doi: 10.3390/molecules27113632 (PMC9182364; doi:10.3390/molecules27113632)
Supplement: Supplementary file 1 [file molecules-27-03632-s001.zip › molecules-1757742-supplementary.pdf]

Supplementary Materials

One-step, Low-Cost, Operator-Friendly and Scalable Procedure to Synthetize Highly Pure N-(4-ethoxyphenyl)-retinamide in Quantitative Yield Without Purification Work-up.

Silvana Alfei* and Guendalina Zuccari*

Department of Pharmacy (DIFAR), University of Genoa, Viale Cembrano, 4, 16148 Genoa, Italy

*alfei@difar.unige.it (S.A.); Tel.: +39 010 355 2296; zuccari@difar.unige.it (G.Z.)

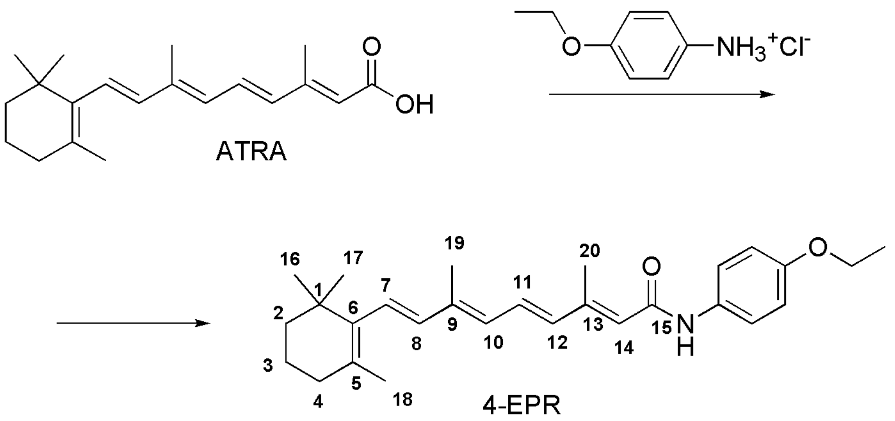

Reaction Summary S1

Yield:63%
1.1 Reagents: Hexachloroacetone , Triphenylphosphine
Solvents: Tetrahydrofuran ; 1 h, 0 °C
1.2 Reagents: Pyridine ; 0 °C-rt; rt
1.3 Reagents: Tetrafluorophthalic anhydride ; 20 - 25 min, rt
1.4 Solvents: Water ; 15 min, rt
1.5 Solvents: Tetrahydrofuran ; 1 h, rt

By: Curley, Robert W., Jr.; et al
World Intellectual Property Organization
WO2003003987 A2 2003-01-16

Reaction Summary S2

Yield: Not available
1.1 Reagents: Oxalyl chloride
Solvents: Et_2O
1.2 Reagents: Degassed dry DMF; mild conditions
Solvents: DMF; rt

By: Maryanoff, Cynthia Anne
European Patent Organization
EP261911 A2 1988-03-30

Reaction Summary S3

Yield: 76%
1.1 Reagents: $SOCl_2$, Pyridine
Solvents: Et_2O
1.2 Reagents:morpholine
Solvents: Et_2O

By:Koenig, H.; et al
From Ger. Offen. (1974), DE 2300107 A1 Jul 11, 1974

Reaction Summary S4

Yield:63%
1.1 Reagents: Triphenylphosphine
Solvents: Tetrahydrofuran
1.2 Reagents: Hexachloroacetone ; 1 h, 0 °C
1.3 Reagents: Pyridine ; 1 - 2 h, 0 °C-rt
1.4 Reagents: Tetrafluorophthalic anhydride
1.5 Solvents: Water
1.6 Reagents: Amberlite A 21

By: Mershon, Serena M.; et al
Bioorg. Med. Chem. Let. (2007), 17(3), 836-840

Reaction Summary S5

Yield: not available
1.1 Reagents: 1-Ethyl-3-(3'-dimethylaminopropyl)carbodiimide hydrochloride
Solvents: Dichloromethane ; 16 h, rt

By: Campos-Sandoval, Jose Angel; et al
J. Med. Chem. (2011), 54(13), 4378-4387

Scheme S1. The three old patents (Reaction Summary S1,S2 and S3) and the generic procedures (Reaction Summary S4 and S5) existing for preparing 4-EPR before our work.

Section S1

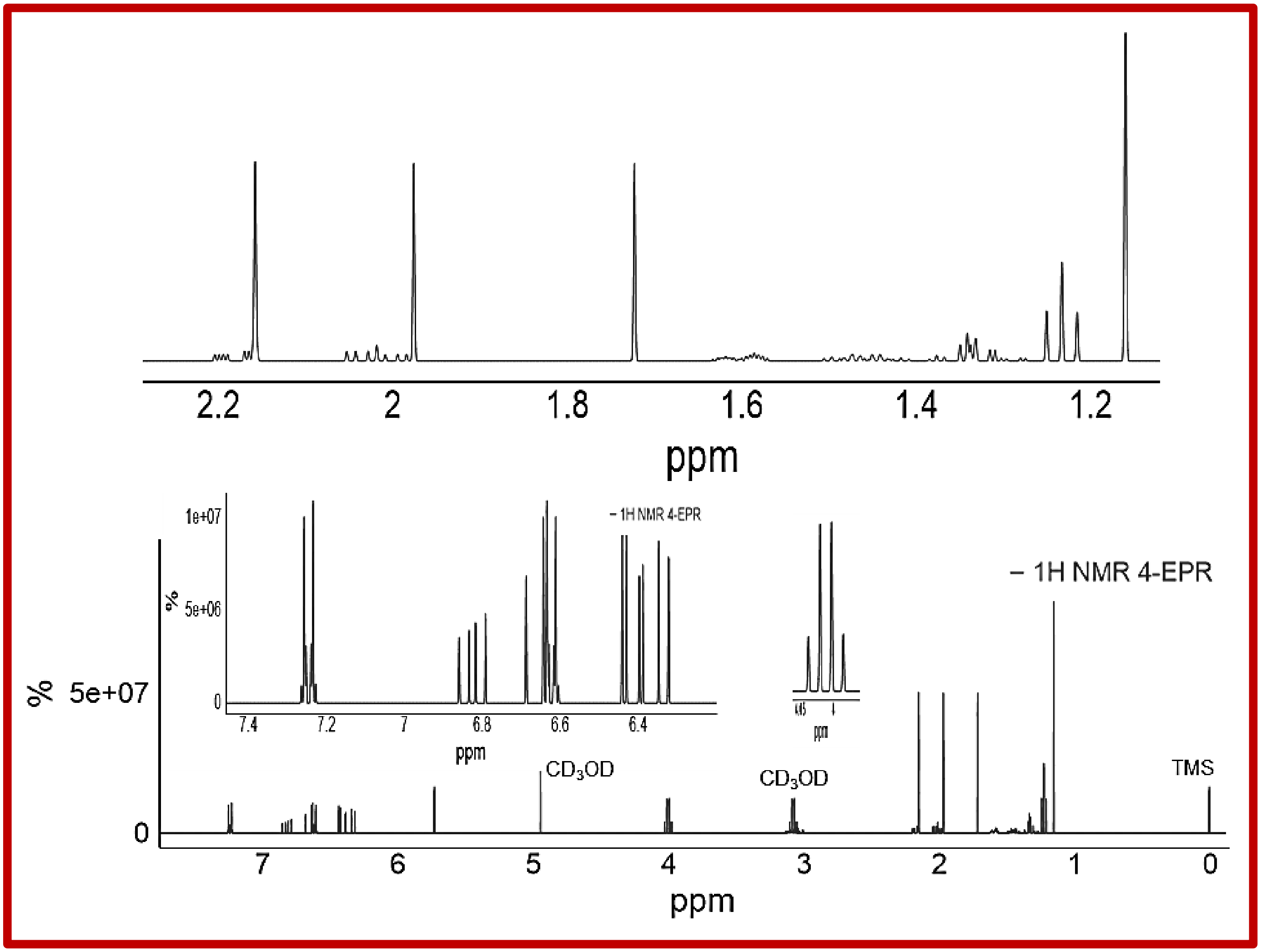

Figure S1a. ^{1}H NMR spectrum with magnifications of significant regions (400 MHz, CD_3OD) of 4-EPR carried out on a Jeol 400 MHz spectrometer (JEOL USA, Inc., Peabody, MA, USA).

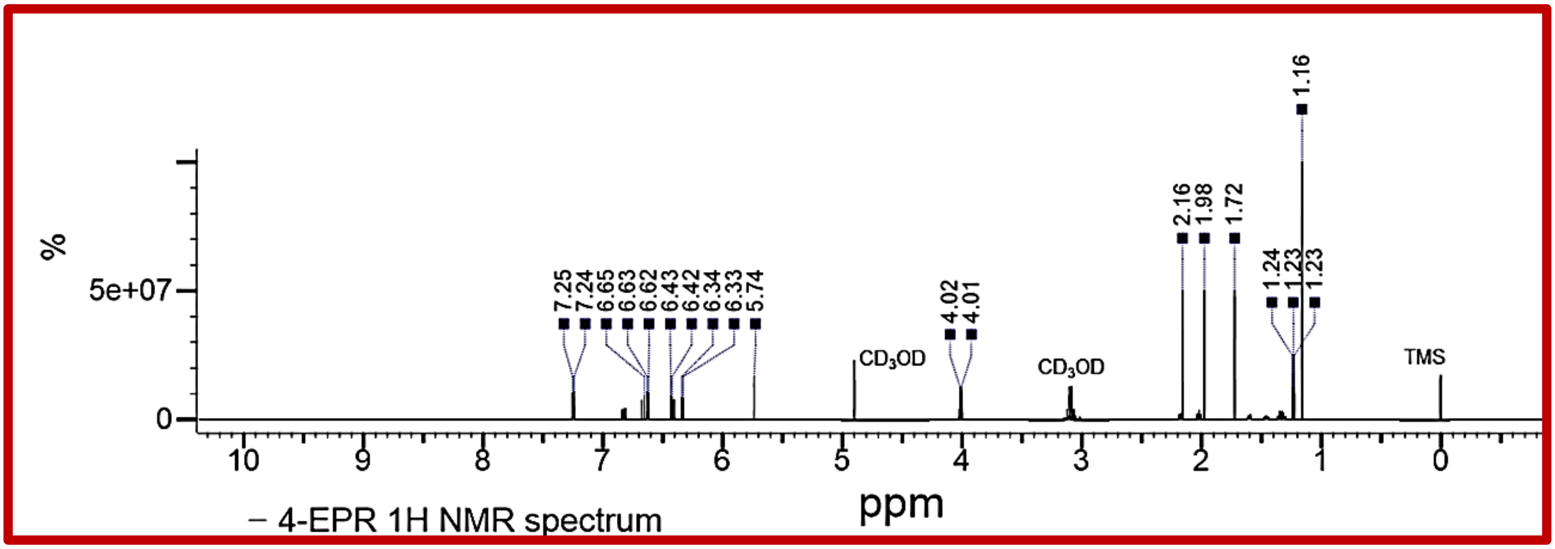

Figure S1b. ^{1}H NMR analysis (400 MHz, CD_3OD) of 4-EPR carried out on a Jeol 400 MHz spectrometer (JEOL USA, Inc., Peabody, MA, USA), including the chemical shifts (ppm) as provided by the instrument.

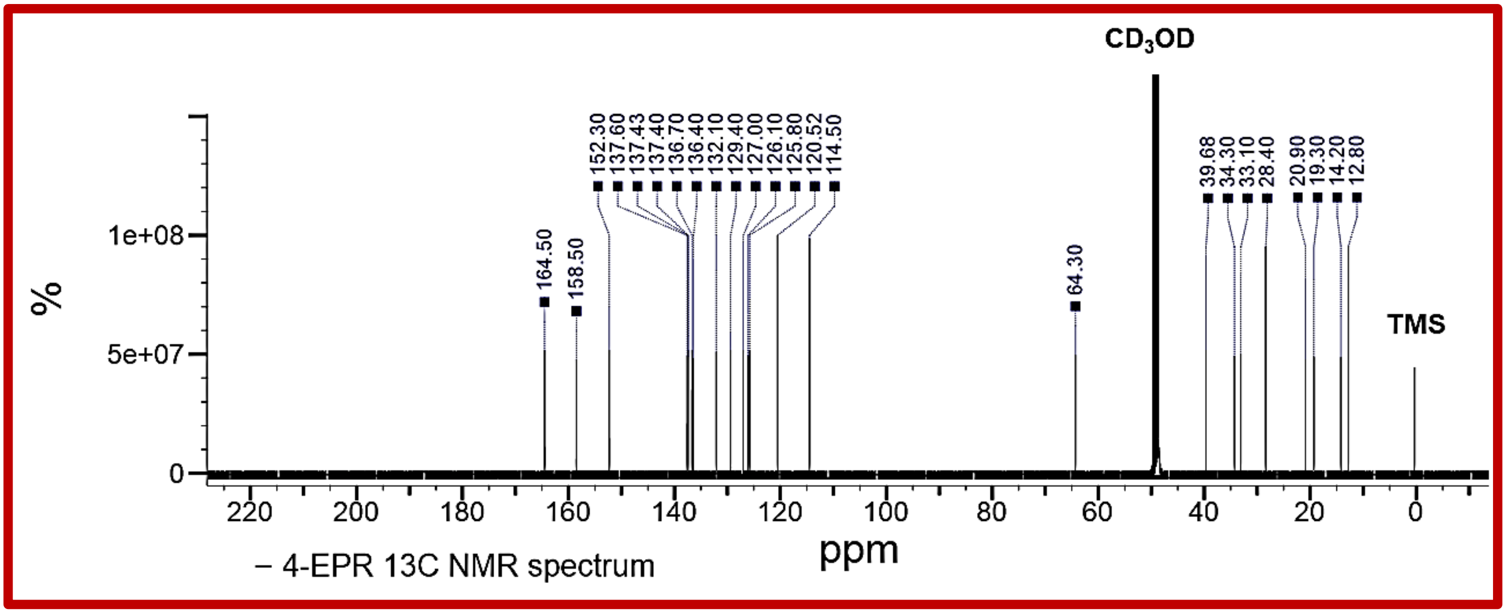

Figure S2. ^{13}C NMR analysis (100 MHz, CD_3OD) of 4-EPR carried out on a Jeol 400 MHz spectrometer (JEOL USA, Inc., Peabody, MA, USA), including the chemical shifts (ppm) as provided by the instrument.

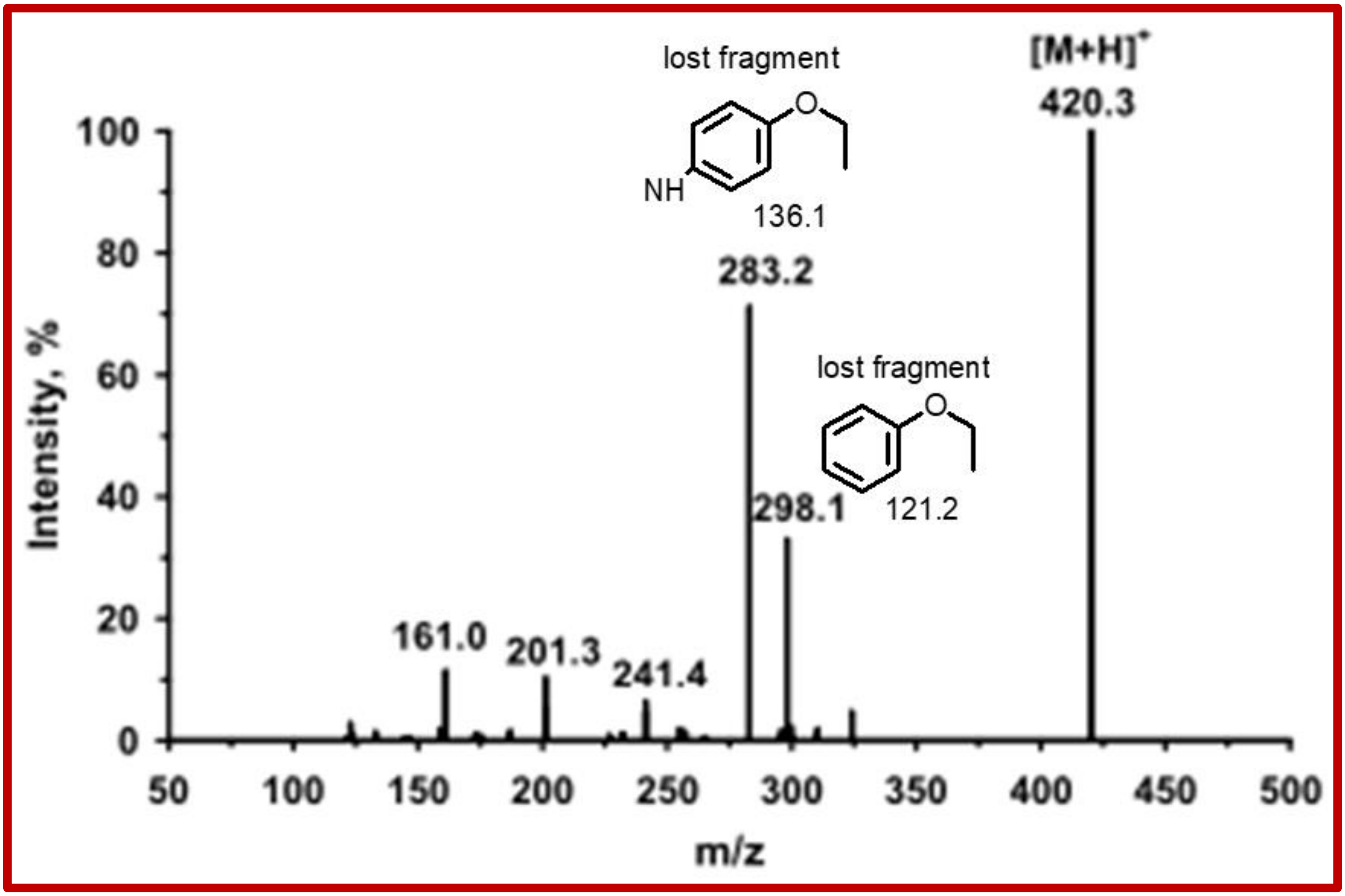

Figure S3. GC-MS spectrum of 4-EPR, obtained with an Ion Trap Varian Saturn 2000 instrument (CI mode, filament current 10 mA) equipped with a DB-5MS (J&W) 30 m, i.d. 0.32 mm, film 1 μm capillary column.

Section S2. Side product (BRAA)

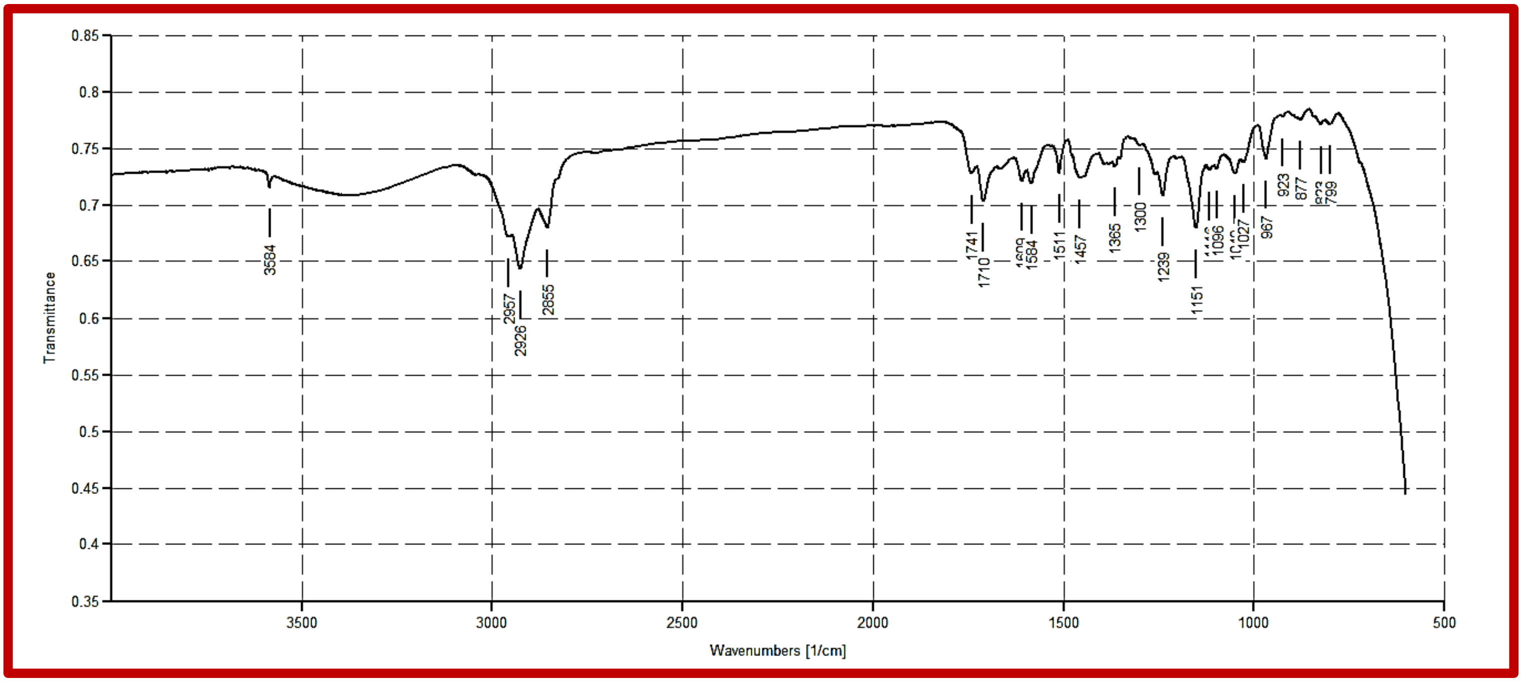

Figure S4. FTIR analysis (KBr) of BRAA, carried out using a Spectrum Two FT-IR Spectrometer (Perkin Elmer, Inc., Waltham, MA, USA). Acquisitions were made from 4000 to 600 cm^{-1}, with 1 cm^{-1} spectral resolution, co-adding 32 interferograms, with a measurement accuracy in the frequency data at each measured point of 0.01 cm^{-1}, due to the internal laser reference of the instrument.
